# A Mode-of-Action Approach for the Identification of Genotoxic Carcinogens

**DOI:** 10.1371/journal.pone.0064532

**Published:** 2013-05-13

**Authors:** Lya G. Hernández, Jan van Benthem, George E. Johnson

**Affiliations:** 1 Laboratory for Health Protection Research, National Institute for Public Health and the Environment (RIVM), Bilthoven, The Netherlands; 2 Institute of Life Science, College of Medicine, Swansea University, Swansea, Wales, United Kingdom; Universität Heidelberg, Germany

## Abstract

Distinguishing between clastogens and aneugens is vital in cancer risk assessment because the default assumption is that clastogens and aneugens have linear and non-linear dose-response curves, respectively. Any observed non-linearity must be supported by mode of action (MOA) analyses where biological mechanisms are linked with dose-response evaluations. For aneugens, the MOA has been well characterised as disruptors of mitotic machinery where chromosome loss via micronuclei (MN) formation is an accepted endpoint used in risk assessment. In this study we performed the cytokinesis-block micronucleus assay and immunofluorescence mitotic machinery visualisation in human lymphoblastoid (AHH-1) and Chinese Hamster fibroblast (V79) cell lines after treatment with the aneugen 17-β-oestradiol (E_2_). Results were compared to previously published data on bisphenol-A (BPA) and Rotenone data. Two concentration-response approaches (the threshold-[Td] and benchmark-dose [BMD] approaches) were applied to derive a point of departure (POD) for *in vitro* MN induction. BMDs were also derived from the most sensitive carcinogenic endpoint. Ranking comparisons of the PODs from the *in vitro* MN and the carcinogenicity studies demonstrated a link between these two endpoints for BPA, E_2_ and Rotenone. This analysis was extended to include 5 additional aneugens, 5 clastogens and 3 mutagens and further concentration and dose-response correlations were observed between PODs from the *in vitro* MN and carcinogenicity. This approach is promising and may be further extended to other genotoxic carcinogens, where MOA and quantitative information from the *in vitro* MN studies could be used in a quantitative manner to further inform cancer risk assessment.

## Introduction

Cancer risk assessment is based on low-dose extrapolation of the risk of chemical carcinogens based on their mode of action (MOA). Genotoxic carcinogens, which are clearly DNA reactive and initiating, follow a linear approach for risk assessment, while indirect and non-DNA reactive carcinogens such as aneugens, and topoisomerase poisons follow a non-linear or threshold approach [1], [2]. Thus establishing the MOA of substances is important for deciding which approach to use for risk characterization. Aneugens are agents which affect cell division and the mitotic spindle apparatus resulting in the loss or gain of whole chromosomes, in comparison to clastogens which are agents that induce breaks in chromosomes leading to sections of the chromosomes being added, deleted or rearranged, or mutagens which are agents which induce mutations. Aneugens were the first class of genotoxic compounds to have well established non-linear dose-responses [3], [4] and the underlying mechanisms responsible for these thresholds are important for hazard and risk assessment. Therefore, distinguishing between aneugens and other genotoxic compounds such as clastogens and mutagens has important implications in cancer risk assessment.

Genotoxicity tests are often used to determine the mutagenic potential of substances because the accumulation of mutations is essential for tumour development, albeit in a qualitative manner. Efforts are presently being made to compare data from *in vitro* and *in vivo* genotoxicity tests and carcinogenicity studies to determine if a quantitative relationship between these two endpoints exists. The main goal here is to investigate whether *in vitro* or *in vivo* genotoxicity tests can provide carcinogenic potency information and whether the concentration-response curves can provide information on genotoxic MOA (linear versus non-linear concentration/dose-response curves observed for clastogens and aneugens, respectively). A recent study showed a correlation between the *in vivo* MN and carcinogenicity for numerous carcinogens with different MOAs [5], and this approach was of interest for the current work. This quantitative framework has gained international interest, particularly with the International Life Science Institute (ILSI) Health and Environmental Sciences Institutes (HESI) *in vitro* genotoxicity testing (IVGT, renamed Genetic Toxicology Technical Committee (GTTC) in 2012) quantitative subgroup which has led to the implementation of different concentration- and dose-response modelling approaches for different compounds [6]. The main objective being to put more emphasis on genetic toxicity data to reduce follow up animal testing and to see if the *in vitro* data can be used in a quantitative fashion [7]. Most of the work to date has focused primarily on DNA reactive genotoxic compounds. Our work is novel in that we aim to apply the various concentration-response approaches on the well-characterised aneugens 17-β-oestradiol (E_2_), bisphenol-A (BPA) and Rotenone.

Several methods are currently available for testing the genotoxic potential of chemicals *in vitro*. The cytokinesis-block micronucleus (CBMN) assay is an accepted test for determining the genotoxic potential of a substance [8]–[11]. Micronuclei (MN) can be formed in dividing cells that either contain chromosome breaks lacking centromeres or whole chromosomes that are unable to travel to the spindle poles during mitosis [12], [13]. The CBMN assay is a convenient and reliable test for the measurement of both chromosome breakage as induced by clastogens and chromosome loss as induced by aneugens. The term aberrant mitotic machinery is defined as the disruption of the microtubules and centrosomes. This can occur by multiple centrosomes being induced by these spindle poisons, resulting in tri, tetra and multi-polar cells, compared to the normal bipolar mitotic cells. Therefore, by visualising the mitotic machinery using immunofluorescence to target α or β-tubulin (microtubules), γ-tubulin (centrosomes) and DNA by using 4',6-diamidino-2-phenylindole (DAPI), possible MOA for aneugens can be determined. This information can be visualised at concentrations surrounding the no-observed effect level (NOEL) for MN induction to obtain a possible MOA for aneuploidy [14], [15]. Here, we put forth an alternative method to the commonly used fluorescence in situ hybridization (FISH) for discriminating between aneugens and clastogens.

In this study we performed the CBMN assay and immunofluorescence mitotic machinery visualisation (IMMV) in the human lymphoblastoid cell line (AHH-1) and the Chinese Hamster fibroblast cell line V79 (V79) exposed to various concentrations of E_2_. Results from E_2_ were compared to previously published data for BPA and Rotenone [14]. Different concentration-response analyses were performed including the threshold-dose (Td) and the benchmark-dose (BMD) approach to determine a POD for *in vitro* MN induction alongside MOA analysis via IMMV. BMD analysis was also performed for carcinogenicity studies to determine an *in vivo* POD. The lowest *in vitro* MN POD was compared to the lowest *in vivo* carcinogenicity POD to investigate whether comparable rankings were observed. This analysis was extended to include 5 additional aneugens (nocodazole, colchicine, mebendazole, carbendazim, and diethylstilbestrol (DES)), 5 clastogens (bleomycin, thiabendazole, chlorambucil, melphalan, and urethane) and 3 mutagens (cytosine arabinoside, 5-fluorouracil and methylmethane sulfonate (MMS)) to see if similar trends were observed between the lowest POD and concentration-response characteristics from the *in vitro* MN assay and *in vivo* carcinogenicity studies.

## Materials and Methods

### Chemicals

All chemicals including cytochalasin-B (CAS number: 14930-962) were purchased from Sigma-Aldrich (Poole, UK) unless otherwise stated. DPX was purchased from Fisher Scientific (Loughborough, UK). Phosphate Buffer Saline (PBS) was prepared using tablets purchased from Sigma, which were dissolved in 1 litre of deionised H_2_0. PBT was prepared using PBS +0.1% Tween 20 (CAS number: 9005-66-7). 17-β-oestradiol (E_2_, CAS number: 50-28-2) was dissolved in dimethylsulphoxide (CAS number: 67-68-5, DMSO).

### Cell Lines

The human lymphoblastoid cell line AHH-1 was obtained from ATCC (CRL-8146, USA, http://www.lgcstandards-atcc.org/Products/All/CRL-8146.aspx) while the Chinese Hamster fibroblast cell line V79 was obtained from European Collection of Cell Cultures (ECACC, 86041102, HPA, UK) (http://www.hpacultures.org.uk/products/celllines/generalcell/detail.jsp?refId=86041102&collection=ecacc_gc).

### *In vitro* Cytokinesis Blocked Micronucleus (CBMN) Assay for E_2_ (Figure 1)

**Figure 1:**
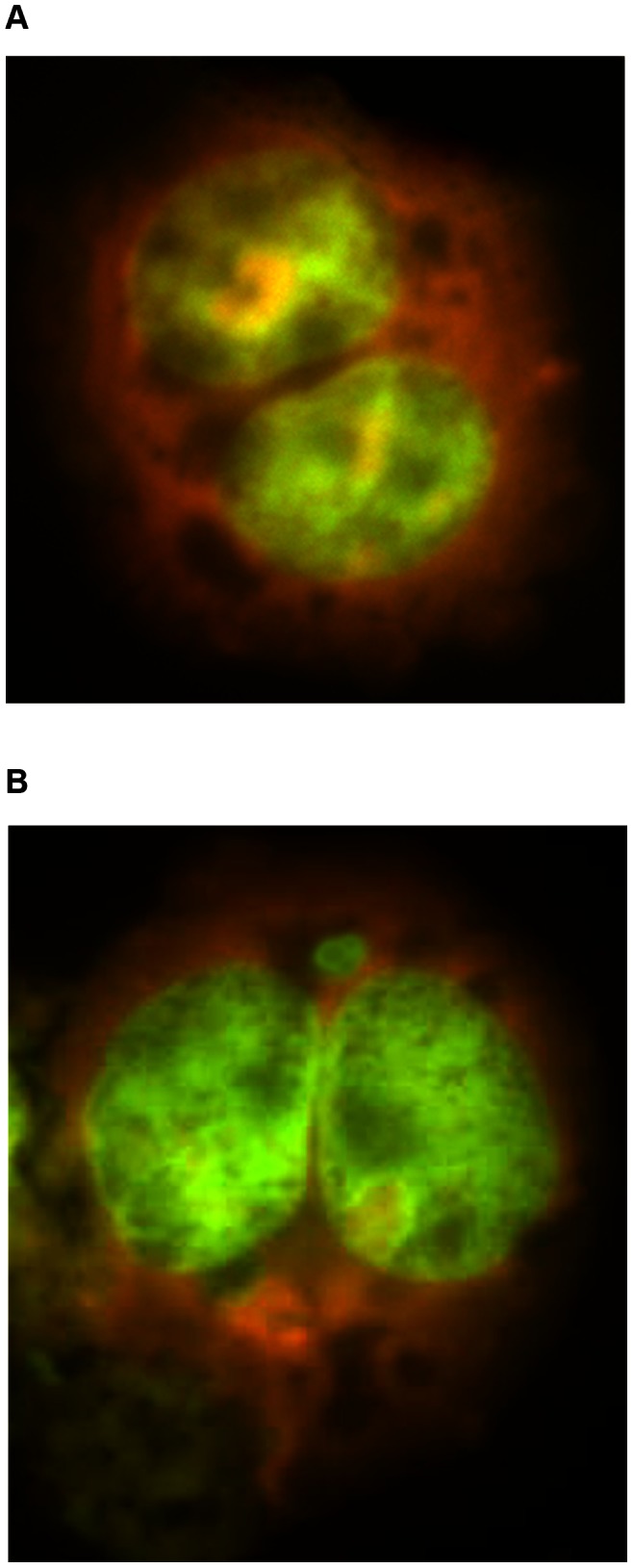
Acridine orange stained AHH-1 cells. (a) Binucleate and (b) binucleate cell with micronucleus, BN-MN.

The *in vitro* CBMN assay in human lymphoblastoid cell line AHH-1 or the Chinese Hamster fibroblast cell line V79 was used to detect both structural and numerical chromosome damage by measuring the formation of MN in interphase cells that have been through a mitotic division [12]. Examples of binucleate cells (BN) with and without MN are shown in Figure 1. In order to determine the effects of E_2_ upon MN induction and chromosome segregation, actively growing cell cultures were exposed to graded concentrations of E_2_ dissolved in DMSO. AHH-1 cells were grown in RPMI 1640 medium (Gibco-Invitrogen, Paisley, UK), 10% horse serum (Gibco-Invitrogen, Paisley, UK) and 1% L-glutamine (Gibco-Invitrogen, Paisley, UK). Cultures were exposed for a complete cell cycle (22 to 26 hours dependent upon any cell cycle delay) in the presence of 3 µg/ml of the actin-inhibitor cytochalasin-B. Cells were washed and centrifuged. Suspensions were then deposited on slides using a cytocentrifuge. This treatment resulted in the production of binucleate cells from those cells that have undergone cell division in the presence of the test chemical and cytochalasin-B. Slides were fixed with methanol and stained with either Giemsa (CAS number: 51811-82-6) or acridine orange (CAS number: 10127-02-3) to detect MN. MN for both control and treated cultures were scored according to previously established criteria [12], [16].

### Aberrant Mitosis Assay: Multiple Centrosomes Induced by E_2_

Sterile glass microscope slides were placed in Petri dishes on which V79 cells were seeded at approximately 7.5×10^4^ cells/ml and grown overnight in fresh medium consisting of Dulbecco’s Modified Eagles Medium (DMEM) without phenol red (Gibco-Invitrogen, Paisley, UK), and supplemented with 10% foetal bovine serum (FBS) (Gibco-Invitrogen, Paisley, UK).Cells were then incubated for 20 hours in the presence of the E_2_ dissolved in DMSO. Colchicine (COL) (CAS number: 64-86-8) was used as a positive control. The highest concentration of E_2_ used did not exceed 50% induced cell toxicity consistent with the OECD guideline (2010) [11].

### Conventional Spindle Staining for E_2_ (Figure 2)

The cells were washed in PBS and then fixed in 3∶1 methanol: acetic acid (CAS number: 64-19-7) (3×14 minutes). Slides were air-dried and then placed in 5% perchloric acid (7601-90-3) at 4°C overnight. 0.5% Brilliant blue (CAS number: 6104-59-4, BB) and 0.5% safranin O (CAS number: 477-73-6, SO) in 15% v/v acetic acid (CAS number: 64-19-7) was added to the slides after washing 10× in distilled water. Slides were air-dried and mounted using DPX (Fisher, Loughborough, UK).

**Figure 2:**
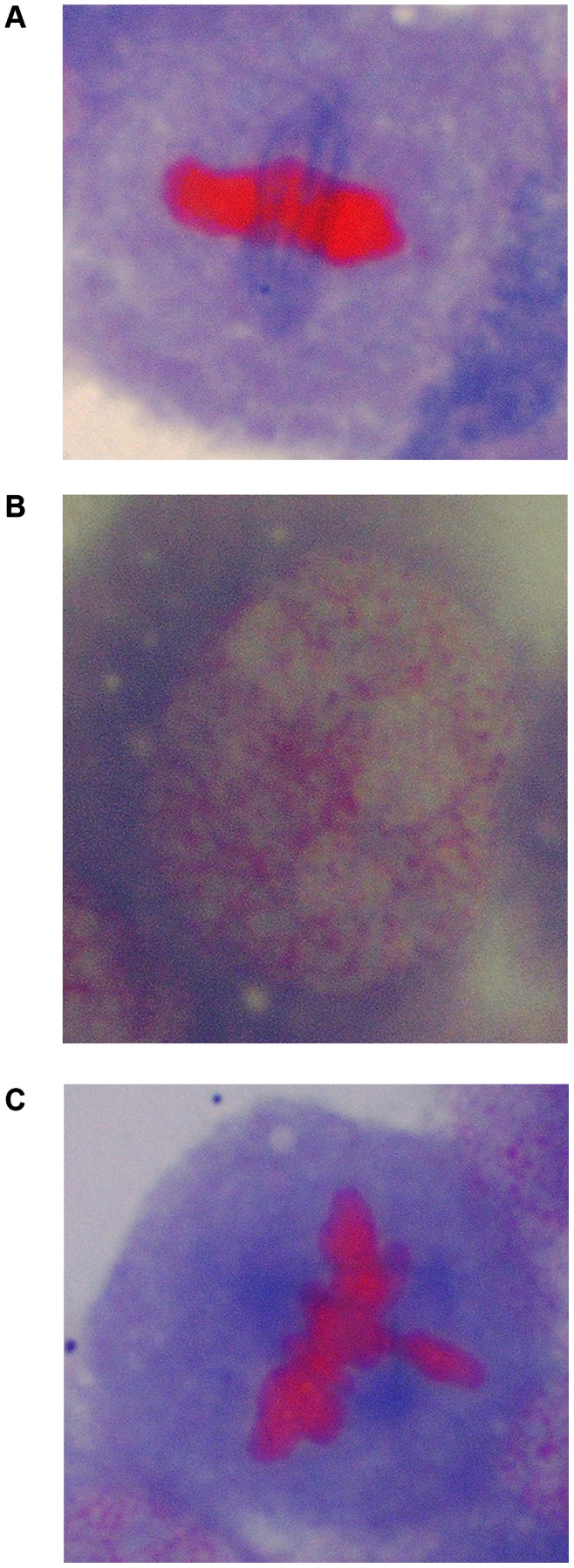
Microtubule staining of normal V79 cells, (a) metaphase and (b) interphase, and an example of a spindle aberration induced by E_2_ in V79 cell, (c) tripolar metaphase. α-Tubulin stains, green = microtubules; γ-tubulin stains, orange = centrosomes; DAPI, blue = chromosomes.

### Immunofluorescence Mitotic Machinery Visualisation (IMMV) for E_2_ (Figure 3)

Cells were washed once in ice-cold PBS, and then fixed for 30 minutes in 90% methanol (CAS number: 67-56-1). Slides were then air dried and stored at −20°C. Frozen slides were placed in 90% methanol at −20°C for 20 minutes and then for 20 s in acetone (CAS number: 67-64-1) at −20°C. Following PBT rinsing, cells were incubated for 2 hours in a humidified chamber at 37°C with a diluted mouse anti γ-tubulin antibody (diluted 1∶200 with PBS) (Sigma, Poole, UK). Then slides were rinsed with PBT and incubated for 2 h at 37°C in a humid chamber with TRITC-conjugated secondary anti-mouse antibody (diluted 1∶32 with PBS). After extensive washing in PBT, cells were kept for 1 h in the presence of a mouse monoclonal anti-α-tubulin conjugate clone (diluted 1∶100 with PBS). DNA was counterstained with DAPI [17], [18].

**Figure 3:**
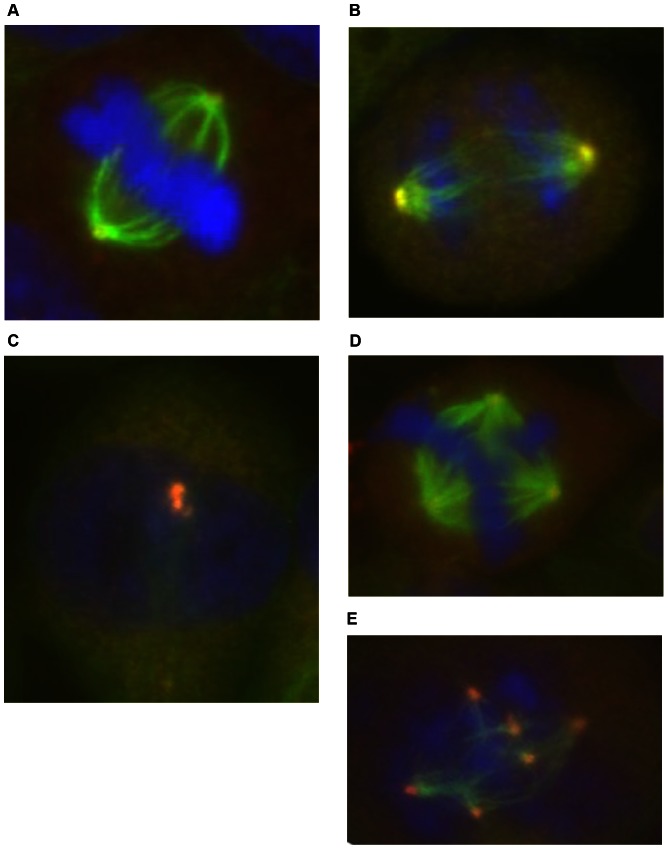
Immunofluorescence mitotic machinery visualisation (IMMV) of normal bipolar V79 cells. (a) Metaphase, (b) telophase and (c) interphase. Examples of spindle aberrations induced by E2 in V79 cell, (d) Tripolar metaphase and (e) multipolar metaphase. α-Tubulin stains, green = microtubules; γ-tubulin stains, orange = centrosomes; DAPI, blue = chromosomes.

### BPA and Rotenone

We have previously characterised the MOA of BPA by employing kinetochore staining [15], [18] which shows if the MN contains a chromosome fragment (*i.e.* compound is clastogenic), or a whole chromosome (*i.e.* compound is aneugenic) [11], [18]. In addition, a summary of NOELs and/or LOELs from genetic toxicity tests after treatment with BPA is illustrated in Table 1.

**Table 1:** Summary of NOELs and/or LOELs from genetic toxicity tests after treatment with BPA [28]–[34].

End Point	Cell Line	NOEL	LOEL	Reference
*In vitro*				
Chromosome aberrations	SHE	200 µM/46 µg/ml		Tsutsui 1998
DNA adducts	SHE		50 µM/11.5 µg/ml	Tsutsui 1998
DNA adducts	Rat liver		100 µM/23 µg/ml	Atkinson and Roy 1995
Chromosome aberrations	CHO	350 µM/80.5 µg/ml	400 µM/92 µg/ml	Hilliard 1998
Chromosome aberrations	CHO	220 µM/50 µg/ml		Ivett 1989
SCE	CHO	130 µM/30 µg/ml		Ivett 1989
Aberrant spindles	V79		100 µM/23 µg/ml	Ochi 1999
γ-tubulin	V79		100 µM/23 µg/ml	Ochi 1999
Multipolar division	V79		100 µM/23 µg/ml	Ochi 1999
Microtubule	Bovine-MT	50 µM/11.5 µg/ml	100 µM/23 µg/ml	Pfeiffer 1997
CMTC	V79		200 µM/46 µg/ml	Pfeiffer 1997
Metaphase arrest	V79	50 µM/11.5 µg/ml	100 µM/23 µg/ml	Pfeiffer 1997
Micronuclei	V79		100 µM/23 µg/ml	Pfeiffer 1997
*In vivo*				
DNA adducts	Rat		200 mg/kg	Atkinson and Roy 1995

NOEL, No-observed effect level; LOEL, lowest-observed effect level; SHE = Syrian Hamster Embryo; SCE, sister chromatid exchange; CHO = Chinese Hamster Ovary; V79 = Chinese Hamster fibroblast cell line; CMTC, cytoplasmic microtubule complex.

### *In vitro* MN Analysis of Other Genotoxic Compounds

An analysis was performed of the literature in search for *in vitro* MN data from different human lymphocyte cell line studies, in addition to the E_2_, BPA and Rotenone derived by Johnson and Parry (2008) [14]. *In vitro* MN data on human lymphocytes exposed to aneugens nocodazole, colchicine, mebendazole, and carbendazim, and the alkylating agent MMS were derived from Elhajouji *et al.* (1997) [3]. *In vitro* MN data on human lymphocytes exposed to aneugens colchicine and DES, the nucleoside analogues cytosine arabinoside and 5-fluorouracil, and the clastogens bleomycin, urethane and thiabendazole were derived from Clare *et al.* (2006) [19]. *In vitro* MN data on human lymphocytes exposed to clastogens chlorambucil and melphalan were derived from Efthimiou *et al.* (2007) [20].

### Derivation of *in vitro* POD from MN Studies

#### Threshold dose approach

*In vitro* MN concentration-response analysis was performed from the data generated in this study for E_2_, and for BPA and Rotenone data derived from Johnson and Parry (2008) [14]. Two methods were used for concentration-response analysis: Td and BMD. Threshold modelling used a similar approach to Gocke and Wall (2009) [21] and Johnson *et al.,* 2009 [22]. This was performed using a 4 step approach. Briefly, Step 1 involved a one-way ANOVA for a dose-related effect (SPSS version 16.0.1). Step 2 involved a comparison of linear and quadratic models using the coefficient of determination (R^2^, SPSS version 16.0.1). The *F* distribution was then used to calculate *P* values in Microsoft Excel 2007. Step 3 involved the determination of no-observed-genotoxic-effect level (NOGEL) or lowest-observed-genotoxic-effect-level (LOGEL) values using a one-sided Dunnett’s test on either untransformed or log-transformed data (SPSS version 16.0.1). Linear and quadratic models were then compared at the NOGEL and below in the same way as described in Step 2. Data that had a flat or zero dose-response slope at the NOGEL and below were then suitable for bilinear or hockey stick analysis. Step 4 involved a comparison of linear versus hockey stick models using the R software package (version 12.2) recommended by Lutz and Lutz (2009) [23]. Parameters, y-intercept, Td, and slope above Td were estimated for best fit of a hockey stick model by minimizing the residual sum of squares. Confidence intervals (CI) were estimated for all parameters using an *F* distribution [23]. If the 95% CI of the derived Td value does not encompass zero, the model is considered a good fit to the data.

#### Benchmark dose approach

The BMD approach was performed using the statistical package PROAST [24] to derive BMC_10_ (*in vitro*) and BMD_10_ (*in vivo*) values for each data set with a benchmark response of 10% as previously done for *in vivo* and *in vitro* genotoxicity data [25]. The BMD approach estimates a dose (i.e., the BMD or BMC) that produces some predetermined, and presumably biologically relevant, increase in the response over control (i.e., the benchmark response). The approach employs mathematical dose–response modeling that takes factors such as sample size and shape of the curve into account [26]. BMC_10_ and BMD_10_ values were derived using the dose-response modeling software package PROAST, developed at the National Institute for Public Health and the Environment (RIVM) in the Netherlands (www.proast.nl). The models used were the exponential models recommended by the European Food Safety Authority [27]. Model selection was performed using the log-likelihood ratio test that assesses whether a statistically significant improvement in the fit is achieved by adding additional parameters. The model with additional parameters is only accepted if the difference in log-likelihoods exceeds the critical value at *P* = 0.05. This is automatically performed in PROAST by selecting the “automatic selection of optimal model from nested family” option. A log-likelihood value is also provided for the “full” model, which is simply the set of the geometric means of the observations at each dose (together with the residual variance). The log-likelihood ratio test can be used to compare the selected model with the full model using a goodness-of-fit test. The model is accepted when the log-likelihood value of the fitted model is significantly better than that of the full model. The BMC_10_ and BMD_10_ with their associated lower (BMDL) confidence limits were then derived from the selected model. Therefore, a BMDL_10_ refers to the estimate of lower 95% CI of a dose that produces a 10% increase over the fitted background level for continuous endpoints, and 10% extra risk for quantal endpoints.

### Derivation of *in vivo* POD for Carcinogenicity

Carcinogenicity data were taken from the National Toxicology Program (NTP) and Carcinogenic Potency Databases (CPD). The BMD approach was used to derive a dose that increases the tumor response by 10% over the modelled control (BMD_10_), with its respective upper (BMDU_10_) and lower (BMDL_10_) confidence limit (Table 2). The lowest confidence limit of the BMD_10_ (BMDL_10_) from the most sensitive tumor endpoint was selected as the POD for carcinogenicity data.

**Table 2:** Analysis of carcinogenicity data from the National Toxicology Program (http://ntp.niehs.nih.gov/).

Compound	Sex	Tissue	BMD_10_ (mg/kg/day)	BMDL_10_ (mg/kg/day)	BMDU_10_ (mg/kg/day)
DES	F	Pituitary gland adenoma	0.001	**0.0005**	0.0024
DES	F	Cervix squamous cell carcinoma	0.07	**0.05**	0.12
DES	M	Testes interstitial cell tumor	0.017	**0.014**	0.02
DES	M	Pituitary gland adenoma	0.003	**0.002**	0.005
DES	F	Pituitary gland adenoma	0.039	**0.03**	0.06
DES	F	Cervix squamous cell carcinoma	0.029	**0.02**	0.04
DES	F	Mammary gland adenocarcinoma, Type B	0.032	**0.011**	0.091
DES	F	Cervix adenoacanthoma	0.086	**0.06**	0.13
DES	M	Testis interstitial cell tumor	0.0066	**0.004**	0.008
DES	F	Mammary gland carcinoma	0.0003	**0.00003**	0.0014
DES	F	Pituitary gland adenoma	0.0009	**0.0004**	0.0019
DES	M	Testis interstitial cell tumor	0.0094	**0.007**	0.012
DES	F	Pituitary gland adenoma	0.0007	**0.0004**	0.0014
E_2_	F	Mammary gland adenocarcinoma	0.56	**0.28**	2.02
BPA	F+M	Leukemia	42.8	**25.99**	114.9
BPA	M	Leukemia	38.5	**20.94**	201.7
BPA	F	Leukemia	56.5	N/A	N/A
Rotenone	M	Parathyroid glad adenoma	N/A	**N/A**	N/A

## Results

### 17-β-oestradiol (E_2_)

**Figure 4:**
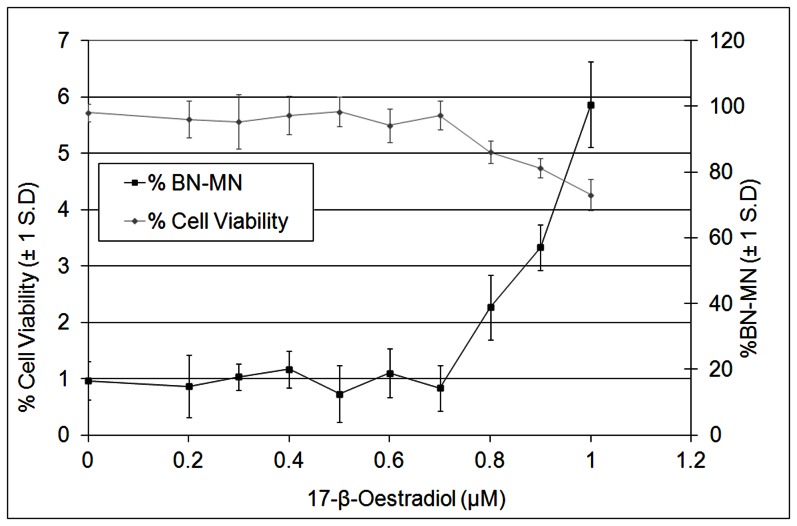
Using the CBMN to assess % binucleate cells with MN (%BN-MN) and cell cytotoxicity and/or cytostasis (% Cell Viability) in AHH-1 cells after E_2_ treatment at super-physiological concentrations. 3×1,000 binucleate cells were examined for the presence of BN-MN. Cell viability (%) was calculated from the cytokinesis-block proliferation index (CBPI) measure (OECD, 2010) by scoring approximately 8,000 cells per dose. 0.8 µM E_2_ and above were significant to *p*<0.05 for BN-MN, by comparison to the control using Dunnett’s.

Aneugenicity, cytotoxicity and cytostasis testing of E_2_ was conducted in AHH-1 cells using the CBMN assay and the aberrant mitosis assay. These endpoints were chosen to observe the genotoxic effects of E_2_ and give a greater understanding of the MOA. E_2_ was found to induce MN at super-physiological levels of E_2_ (0.8–1.0 µM) with a significant decrease in cell viability (*p*<0.05) at the same concentrations (Figure 4). The td-L-CI for MN induction was observed at 0.74 µM and for effects on spindle formation (Tripolar) at 0.45 µM (Table 3). The first significant (*p*<0.05) increase (LOEL) for MN induction and for effects on spindle formation (Tripolar) was observed at 0.8 µM. The BMD approach showed a BMCL_10_ of 0.40 and 0.02 µM for MN and spindle formation induction, respectively (Table 3). If there was a decrease of 50% cell viability or less at a genotoxic (*i.e.* clastogenic) effect, then the MOA was said to potentially be a cytotoxicity related secondary mechanism and not a true genotoxic response [11]. However, there was only a decrease of 10%–20% cytotoxicity and/or cytostasis when a 2–3× fold increase in MN is observed, which indicated that E2 was genotoxic through a non-cytotoxicity related MOA. The NOEL was defined as the lowest value produced between the Td-L-CI and BMCL10 in both the chromosome loss (MN) and spindle formation effects. This was justified because non-disjunction is known to occur at lower concentrations than chromosome loss ([4](Table 3). With this criterion, the NOEL for E_2_ was 0.02 µM. The most sensitive carcinogenicity endpoint with the lowest BMDL_10_ was observed in the mammary gland with a BMDL_10_ of 0.28 mg/kg/day (1.03 µM/day; Tables 2 and 3).

**Table 3:** Summary of the BMDL_10_ from carcinogenicity studies, and td-L-CI and BMCL_10_ values from AHH-1 and the Chinese Hamster fibroblast cell line V79 cells exposed to E_2_, BPA and Rotenone.

Compound	CP, Most sensitive tissue	CP, BMDL_10_ (mg/kg/day)	CP, BMDL_10_ (µM/day)	POD *in vitro* (µM)	*In vitro,* td-L-CI (µM) MI	*In vitro,* td-L-CI (µM) MN	*In vitro,* BMCL_10_ (µM) MI	*In vitro,* BMCL_10_ (µM) MN
**DES**	mammary gland carcinoma	3.00 E-05	1.1 E-04					
**E_2_**	mammary gland adenocarcinoma	0.28	1.03	0.02	0.48	0.74	0.42	0.40
**BPA**	Leukaemia	20.94	91.73	2.58	30.39	2.58	29.93	5.48
**Rotenone**	parathyroid gland adenoma?	no DR	no DR	no CR	1.24 E-04	N/A	5.32E-06	no CR

POD is defined as lowest Td-L-CI and BMCL10 in MN or spindle formation effects. µM/day were calculated from mg/kg/day, assuming 1 kg = 1 litre. CP, carcinogenic potency was derived from studies carcinogenic potency database (www.berkley.org) and National Toxicology Program (NTP); POD, point of departure; MI, mitotic index; MN, frequency of micronuclei formation, DR, dose-response; CR, concentration response.

## Discussion

The goal of this analysis was to investigate whether carcinogenic potency information (i.e. cancer potency ranking) could be derived from *in vitro* MN data. For this, several quantitative dose-response methods were investigated for the selection of a suitable *in vitro* MN POD for BPA, E_2_, and Rotenone. In order to investigate which method was more appropriate for POD derivation, the traditional method for analysing *in vitro* genotoxicity data (i.e. derivation of no-observed-effect-level (NOEL) or a lowest-observed-effect-level (LOEL)) was compared to more recent quantitative methods. First, a summary of BPAs effects in different genetic toxicology tests is represented in Table 1. From Table 1, it was clear that the NOELs vary significantly between the different *in vitro* genotoxic endpoint ranging from 25 to 250 µM. The NOEL or LOEL are not the ideal method for performing concentration-response analysis. Comparison of the NOELs from chromosomal aberrations in Chinese Hamster Ovary (CHO) cells between Hilliard *et al.* (1998) [28]350 µM and Ivett *et al.* 1989 [29] 220 µM clearly demonstrates how deriving the NOEL using the traditional method is highly dependent on experimental conditions. In addition, NOEL only used one concentration and not the entire data set, and no confidence limits can be derived. In contrast, quantitative methods such as the Td and BMD approach use all the data, are not so dependent on experimental conditions and provide confidence limits [26]. For this reason, the Td and BMD approach were selected for the derivation of POD from *in vitro* MN studies. The lowest reported NOEL observed was 50 µM or 11.5 µg/ml for metaphase arrest in the V79 cell line (Table 1) [30]. For the current study in which we carried out extensive statistical modelling on our previously published BPA data [14], the td-L-CI for MN-induction and disruption in spindle formation was observed at 2.58 and 15.42 µM, respectively (Table 3). Similarly, BMCL_10_ were 5.48 and 3.13 for MN and spindle effects, respectively. Therefore, the POD for *in vitro* MN for BPA was 2.58 µM.

**Figure 5:**
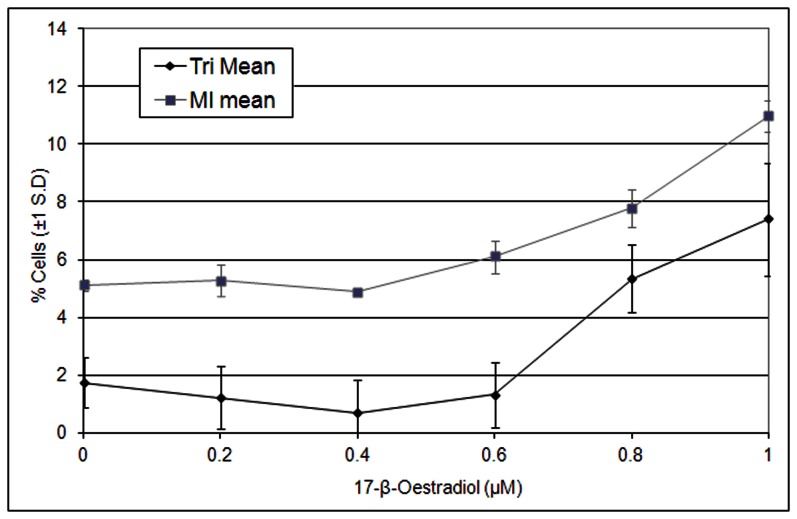
Using the Aberrant Mitosis Assay to show the activity of 17-β-oestradiol (E_2_) as a spindle poison, and to give the concentration-response relationship at super-physiological concentrations. Tripolar (Tri) was calculated using number of tripolar cells compared to number of mitotic cells (3×100 in total) using IMMV. Mitotic Index (MI) was calculated using number of mitotic cells compared to number of interphase cells (3×1,000 cells in total) using conventional spindle staining. 0.8 µM E_2_ and above were significant to *p*<0.05 for both MI and Tri, by comparing to the control using Dunnett’s.

**Figure 6:**
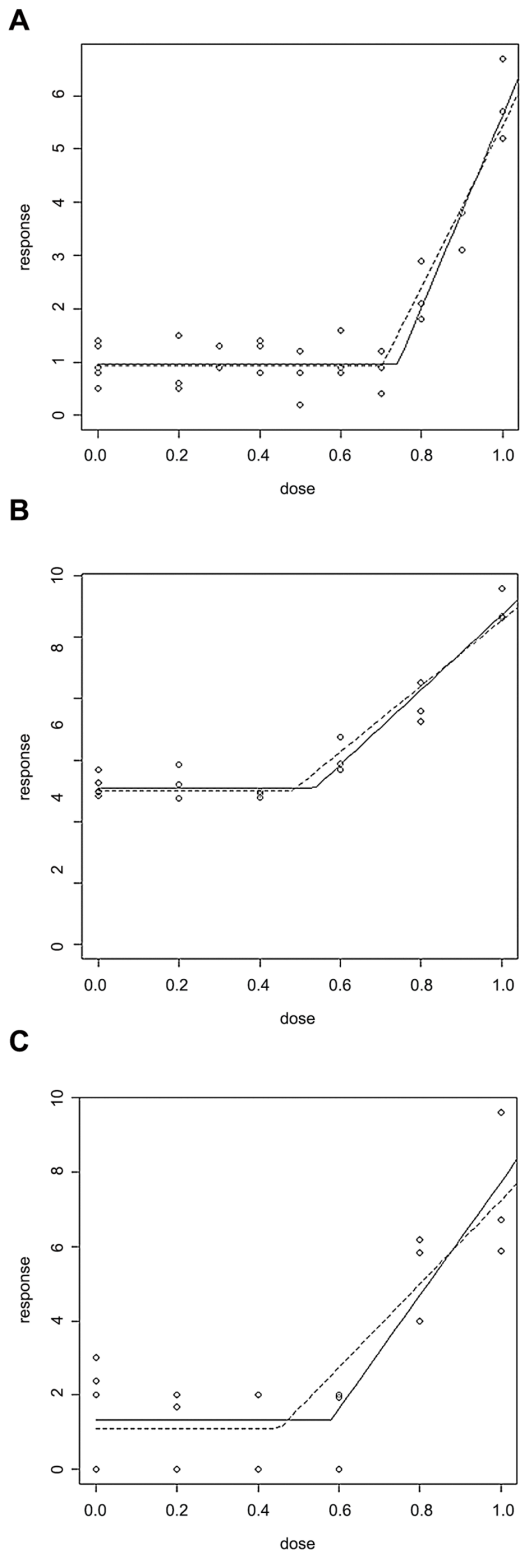
Mammalian cells treated with 17-β-oestradiol (E_2_) treated mammalian cells with the following endpoints. (a) MN (AHH-1), (b) MI (V79) and (c) Tripolar (V79). Graphs shown are from the Lutz and Lutz (2009, [23]) hockey stick model for R, with the dotted line being the lower 95% confidence interval. The x-axis ‘dose’ is in µM, and y-axis ‘response’ is % cells.

**Figure 7:**
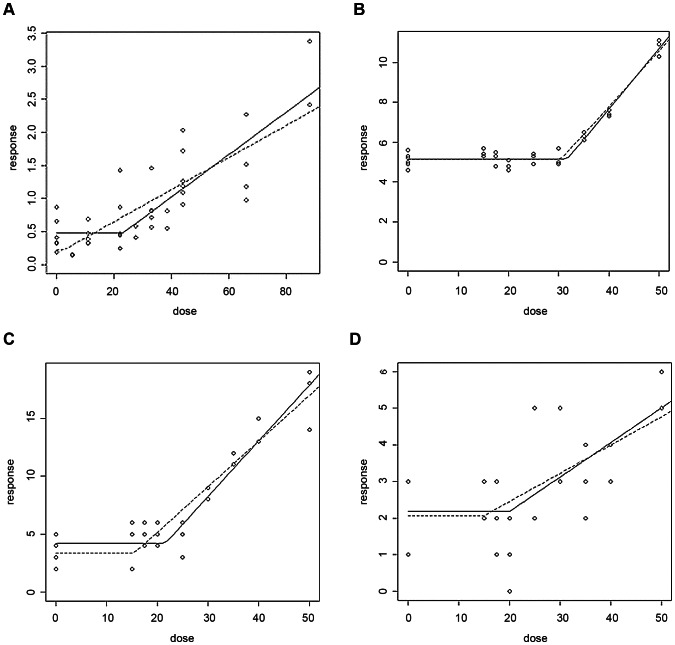
Mammalian cells treated with bisphenol-a (BPA) using the following endpoints. (a) MN (AHH-1), (b) MI (V79) and (c) Tripolar (V79), and (d) multi+tetrapolar (V79). Graphs shown are from the Lutz and Lutz (2009, [23]) hockey stick model for R, with the dotted line being the lower 95% confidence interval. The x-axis ‘dose’ is in µM, and y-axis ‘response’ is % cells.

E_2_ and BPA (xenoestrogens) are spindle poisons with well-characterised thresholds for genotoxic activity [3], [4], while no concentration-response was observed with Rotenone (Table 3). E_2_ and BPA showed clear thresholds for MN, mitotic index (MI) and tripolarity. This is to be expected as hormones are presumed to have non-linear concentration and dose-responses [1], and this study confirms these observations (Figures 4–7). In addition, comparisons of PODs between *in vitro* MN and carcinogenicity were made. Table 3 demonstrated that the BMCL_10_s for *in vitro* MN were ranked as E_2_>BPA>>Rotenone. The carcinogenicity ranking of the most sensitive tumour endpoint (Table 2) was also E_2_>BPA>>Rotenone (Table 3). These results, although with limited number of compounds, were very promising indicating the potential for deriving carcinogenic potency information from *in vitro* MN studies.

**Figure 8:**
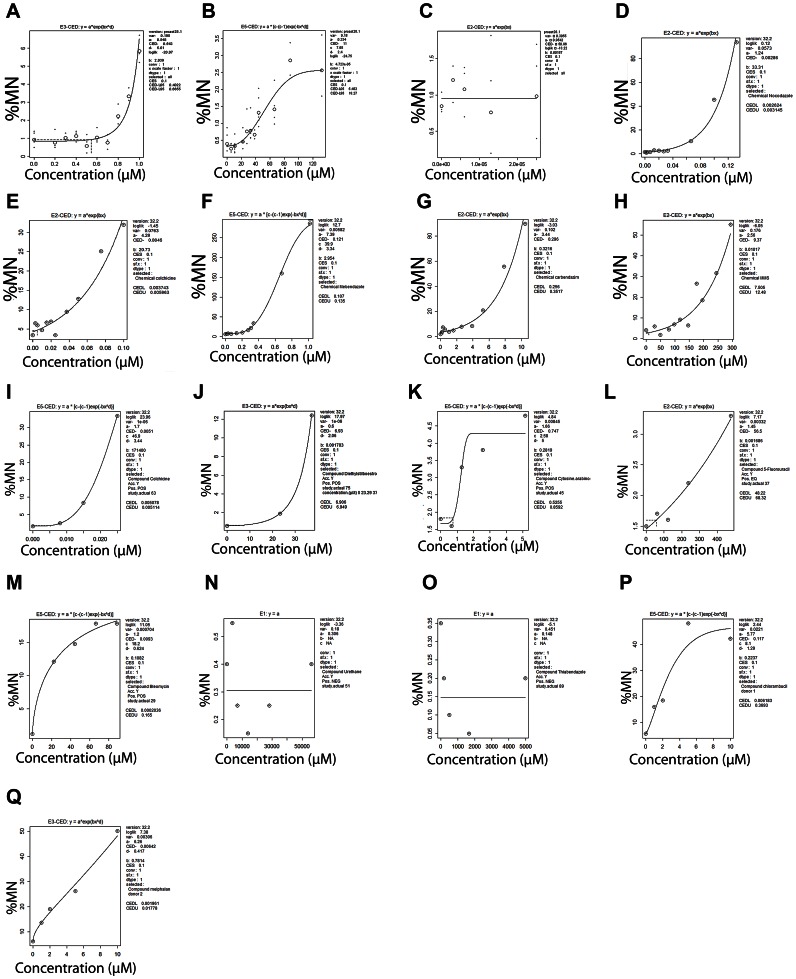
BMD analysis of studies by Johnson and Parry (2008, [14]) for (a) E2, (b) BPA and (c) Rotenone; by Elhajouji et al. (1997, [3]) (d) nocodazole, (e) colchicine, (f) mebendazole, (g) carbendazim, and (h) MMS; by Clare et al. (2006 [19]) for (i) colchicine, (j) DES, (k) cytosine arabinoside, (l), 5-fluorouracil, (m) bleomycin (n) urethane, and (o) thiabendazole; and by Efthimiou et al. (2007, [20]) for (p) chlorambucil and (q) melphalan.

Given the promising ranking results observed with E_2_, BPA and Rotenone, we extended our analysis and performed a literature search for *in vitro* MN data in human lymphocytes [3], [14], [19], [20]. With this analysis we wanted to explore the applicability of using concentration-response analysis to extrapolate information in regards to linear *versus* non-linear concentration-responses and carcinogenic potency. Based on the concentration-response curves in Figures 8a–q, a clear distinction in the shape of the concentration-response curves from *in vitro* MN for aneugens (Figure 8: a, E_2_; b, BPA; c, Rotenone; d, nocodazole; e, colchicine; f, mebendazole; g, carbendazim; i, colchicine; and j, DES) and clastogens (Figure 8: m, bleomycin; p, chlorambucil; and q, melphalan). The *in vitro* MN concentration dose-response curves were clearly non-linear for aneugens and linear from clastogens. Substances which were more mutagenic than clastogenic and require metabolic activation such as urethane (Figure 8 n) showed no concentration-response. The concentration-response curves from the *in vitro* MN from mutagenic substances such as methyl methanse sulfonate (MMS; Figure 8 h) and cytosine arabinoside (Figure 8 k) seemed to have non-linear concentration-response curves. This has been previosly demonstrated for MMS [25] but more research on the MOA of cytosine arabinoside is needed to verify our observation. Other genotoxic substances such as 5-fluorouracil (Figure 8 l) had concentration-response curves which were clearly linear at the concentrations tested. Thus, similar concentration-response curves could be used to group substances in terms of their genotoxic MOA and further obtain potency information.

In terms of carcinogenic potency, it was very difficult to make any inferences given the limited *in vitro* MN and carcinogenicity data (Figure 8 a–q). For the study of Elhajouji *et al.* (1997) [3], only carbendazim had carcinogenicity data. The study by Clare *et al.* (2006) [19] showed a carcinogenicity ranking of BMDL_10_a of DES>urethane>5-fluorouracil. The BMCL_10_s from *in vitro* MN showed a genotoxicity ranking of bleomycin>colchicine>cytosine arabinoside>DES>5-fluorouracil (Table 4). For compounds which had both MN and carcinogenicity data, the rankings did not differ significantly, with the exception of urethane which requires metabolic activation and had no concentration-response. For the study by Efthimiou *et al.* (2007) [20], the carcinogenicity ranking was chlorambucil>melphalan while the genotoxicity ranking was melphalan>chlorambucil. No true conclusions can be made until more substances are tested. To the best of our knowledge, this is the firt study to try to attempt to investigate whether carcinogenic potency information can be derived from *in vitro* MN studies in human lymphocytes using quantitative approaches. Atlhough inconclusive, these results were promising and more *in vitro* MN studies under the same conditions (treatment schedule and recovery) and carcinogenicity studies are needed.

**Table 4:** Summary of BMDL_10_s derived from different human lymphocyte and AHH-1 cell line studies [3], [14], [19], [20].

Compound	Classification	CP, Most sensitive tissue/endpoint	CP,BMDL_10_(µM/day)	*In vitro,* BMCL_10_ (µM) MN (%MN)	*In vitro* cell line	Reference
**E_2_**	aneugen	mammary gland adenocarcinoma	1.03	0.40	AHH-1 cell line	Johnson and Parry (2008)
**BPA**	aneugen	leukemia	91.73	5.48	AHH-1 cell line	Johnson and Parry (2008)
**Rotenone**	aneugen	parathyroid gland adenoma?	no DR	no CR	AHH-1 cell line	Johnson and Parry (2008)
**Nocodazole**	aneugen	N/A	N/A	0.0026	human lymphocytes	Elhajouji et al. (1997)
**Colchicine**	aneugen	promoter in two-stage skin tumor model	N/A	0.004	human lymphocytes	Elhajouji et al. (1997)
**Mebendazole**	aneugen	N/A	N/A	0.107	human lymphocytes	Elhajouji et al. (1997)
**Carbendazim**	aneugen	hepatocellular adenomas and carcinomas	62.08	0.26	human lymphocytes	Elhajouji et al. (1997)
**MMS**	alkylating agent	N/A	*N/A*	7.51	human lymphocytes	Elhajouji et al. (1997)
**Colchicine**	aneugen	N/A	N/A	0.005	human lymphocytes	Clare et al. (2006)
**DES**	aneugen	mammary gland carcinoma	0.0001	6.90	human lymphocytes	Clare et al. (2006)
**Cytosine arabinoside**	nucleoside analogue	N/A	N/A	0.53	human lymphocytes	Clare et al. (2006)
**5-Fluorouracil**	nucleoside analogue	lung and lymphoreticular system	22.76	48.20	human lymphocytes	Clare et al. (2006)
**Bleomycin**	clastogen	N/A	N/A	0.0002	human lymphocytes	Clare et al. (2006)
**Urethane**	clastogen (requires metabolic activation)	lung alveolar-bronchiolar adenoma	0.11	no CR	human lymphocytes	Clare et al. (2006)
**Thiabendazole**	clastogen	no positive in CPD	no DR	no CR	human lymphocytes	Clare et al. (2006)
**Chlorambucil**	clastogen	Lymphosarcoma	0.0007	0.006	human lymphocytes	Efthimiou et al. (2007)
**Melphalan**	clastogen	tumor-bearing animals mixed	0.013	0.002	human lymphocytes	Efthimiou et al. (2007)

CP, carcinogenic potency was derived from studies carcinogenic potency database (www.berkley.org) and National Toxicology Program (NTP); POD, point of departure; MI, mitotic index; MN, frequency of micronuclei formation, DR, dose-response; CR, concentration response.

### Conclusions

Here we demonstrated that combining the micronucleus assay along with aberrant mitotic analysis in AHH-1 and V79 cells, has risk assessment applications for the identification of aneugens, and the derivation of PODs using Td and BMD statistical modelling approaches. The traditional NOEL method for deriving POD is less suitable for analyzing *in vitro* genotoxicity data and quantitative approaches such as the Td and BMD are recommended for future POD derivation. The concentration-response curves from the *in vitro* MN in AHH-1 and human lymphocytes provide useful information on linear versus non-linear concentration-response which has risk assessment implications. Comparison of POD ranking between the *in vitro* MN and carcinogenicity were comparable with E_2_, BPA and Rotenone but comparisons with other clastogens and mutagens were inconclusive.

Further analysis is needed to investigate whether POD derivation from *in vitro* MN studies may provide carcinogenic potency information.
